# CNOT2 Is Critically Involved in Atorvastatin Induced Apoptotic and Autophagic Cell Death in Non-Small Cell Lung Cancers

**DOI:** 10.3390/cancers11101470

**Published:** 2019-09-30

**Authors:** Jihyun Lee, Ji Hoon Jung, Jisung Hwang, Ji Eon Park, Ju-Ha Kim, Woon Yi Park, Jin Young Suh, Sung-Hoon Kim

**Affiliations:** College of Korean Medicine, Kyung Hee University, Seoul 02447, Korea; jhlee096@naver.com (J.L.); johnsperfume@gmail.com (J.H.J.); hjsung0103@naver.com (J.H.); wdnk77@naver.com (J.E.P.); 964juha@daum.net (J.-H.K.); wy1319@naver.com (W.Y.P.); jysuh3670@gmail.com (J.Y.S.)

**Keywords:** atorvastatin, non-small lung cancer cells, apoptosis, autophagy, CNOT2

## Abstract

Though Atorvastatin has been used as a hypolipidemic agent, its anticancer mechanisms for repurposing are not fully understood so far. Thus, in the current study, its apoptotic and autophagic mechanisms were investigated in non-small cell lung cancers (NSCLCs). Atorvastatin increased cytotoxicity, sub G1 population, the number of apoptotic bodies, cleaved poly (ADP-ribose) polymerase (PARP) and caspase 3 and activated p53 in H1299, H596, and H460 cells. Notably, Atorvastatin inhibited the expression of c-Myc and induced ribosomal protein L5 and L11, but depletion of L5 reduced PARP cleavages induced by Atorvastatin rather than L11 in H1299 cells. Also, Atorvastatin increased autophagy microtubule-associated protein 1A/1B-light chain 3II (LC3 II) conversion, p62/sequestosome 1 (SQSTM1) accumulation with increased number of LC3II puncta in H1299 cells. However, late stage autophagy inhibitor chloroquine (CQ) increased cytotoxicity in Atorvastatin treated H1299 cells compared to early stage autophagy inhibitor 3-methyladenine (3-MA). Furthermore, autophagic flux assay using RFP-GFP-LC3 constructs and Lysotracker Red or acridine orange-staining demonstrated that autophagosome-lysosome fusion is blocked by Atorvastatin treatment in H1299 cells. Conversely, overexpression of CCR4-NOT transcription complex subunit 2(CNOT2) weakly reversed the ability of Atorvastatin to increase cytotoxicity, sub G1 population, cleavages of PARP and caspase 3, LC3II conversion and p62/SQSTM1 accumulation in H1299 cells. In contrast, CNOT2 depletion enhanced cleavages of PARP and caspase 3, LC3 conversion and p62/SQSTM1 accumulation in Atorvastatin treated H1299 cells. Overall, these findings suggest that CNOT2 signaling is critically involved in Atorvastatin induced apoptotic and autophagic cell death in NSCLCs.

## 1. Introduction

Though non-small cell lung cancers (NSCLCs), approximately 85% of all lung cancers, have been treated for recent decades by multimodality treatments consisting of extended surgical resection, radiotherapy and chemotherapy, a five-year survival rate is still considered below 20% in NSCLC with multiple mutations, including KRAS and epidermal growth factor receptor (EGFR) [1,2]. Hence, recently molecular target therapy is considered an attractive strategy for treatment of NSCLC [3].

Recently induction of cell death has been regarded a promising strategy for effective cancer therapy [4]. Generally programmed cell death includes apoptosis (Type I cell death), autophagic cell death (Type II cell death) [5,6]. Autophagy, which implies “self-eating”, is a ubiquitous process that occurs in all eukaryotic cells [7,8,9]. It is a complex catabolic process, in which double membrane vesicles (autophagosomes) engulf large intracellular components, such as organelles or proteins, and then fuse with lysosomes to degrade its contents. There are three defined types of autophagy such as micro-autophagy [10], macroautophagy [11], and chaperone-mediated autophagy [11,12]. In terms of survival mechanism of autophagy, once autophagy response is induced, the entire autophagic flux is completed in progress and then the homeostasis of the cell is maintained. In contrast, impaired autophagy blocks the fusion of autophagosome and lysosome leading to cell death [13].

CCR4-NOT complex (CNOT) consists of eleven subunits as a master regulator of mRNA stability, transcription, and translation and mRNA export [14]. Among them, human CNOT2 is known to regulate the deadenylase activity and structural integrity of the CCR4–NOT complex. CNOT2 was reported to be closely associated with p62/SQSTM1-degradation as an autophagy negative regulator [15]. CNOT2 depletion destabilized the CCR4–NOT complex. Furthermore, CNOT2 depletion caused apoptosis in a caspase-dependent manner [16].

Atorvastatin, one of the statin class inhibitors, is known to treat hypercholesterolemia through the inhibition of 3-hydroxy-3-methylglutaryl-coenzyme A (HMG-CoA) reductase, and recently inhibit tumor growth in various cancers [17,18]. Furthermore, Atorvastatin was shown to inhibit myocardial Rac1-GTPase activity, and in turn suppress the NADPH (nicotinamide adenine dinucleotide phosphate) oxidase activity and ROS generation, thereby, beneficial for patients with chronic heart failure. Also, inhibition of autophagy was reported to potentiate Atorvastatin-induced apoptotic cell death in human bladder cancer cells [19]. Thus, in the current study, the apoptotic and autophagic cell death mechanisms of Atorvastatin were investigated in A549, H596, H460, and H1299 NSCLCs in association with CNOT2 signaling.

## 2. Results

### 2.1. Effects of Atorvastatin on Cytotoxicity in H596, H460, and H1299 Cells

**Figure 1:**
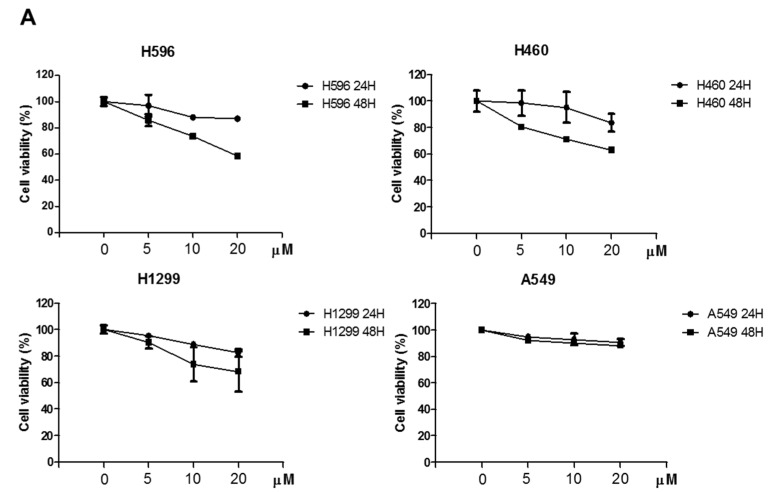

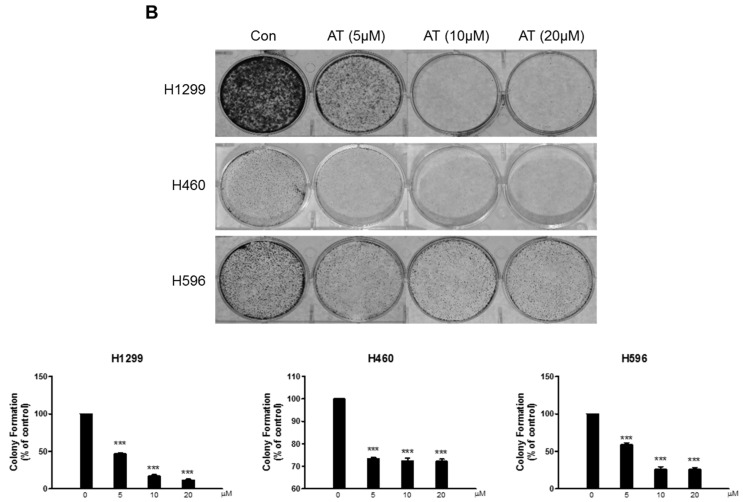
Cytotoxic and anti-proliferative effects of Atorvastatin in non-small cell lung cancers (NSCLCs). (**A**) Cytotoxicity of Atorvastatin in the H596, H460, H1299, and A549 cells. Four lung cancer cell lines were treated with various concentrations of Atorvastatin (0, 5, 10, and 20 μM) for 24 or 48 h. Then cell viability was determined by MTT assay. (**B**) Anti-proliferative effect of Atorvastatin in the H596, H460, and H1299 cells by colony formation assay. Data represent means ± SEM of triplicate samples. *** *p* < 0.001 vs. untreated control.

Cytotoxicity of Atorvastatin (AT) was evaluated in A549, H596, H460, and H1299 cells by MTT assay (Figure 1A). Atorvastatin induced cytotoxicity in H596, H460, and H1299 cells compared with A549 cells and also was confirmed to inhibit the number of colonies in H596, H460, and H1299 cells by colony formation assay (Figure 1B). Herein H1299 cells were found most susceptible to Atrovastatin among 3 NSCLCs compared to A549 cells. Hence, we performed further mechanism experiments on apoptosis and autophagy mainly in H1299 cells.

### 2.2. Atorvastatin Induced Apoptosis via Ribosomal Protein L5 and L11 in NSCLCs

**Figure 2:**
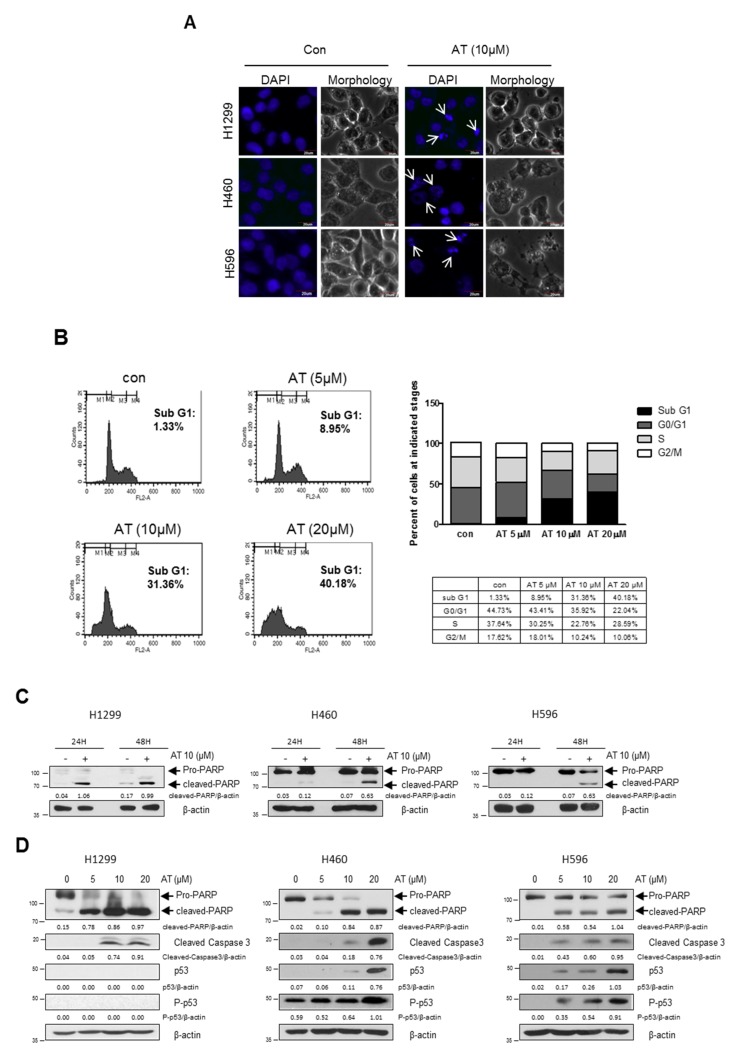

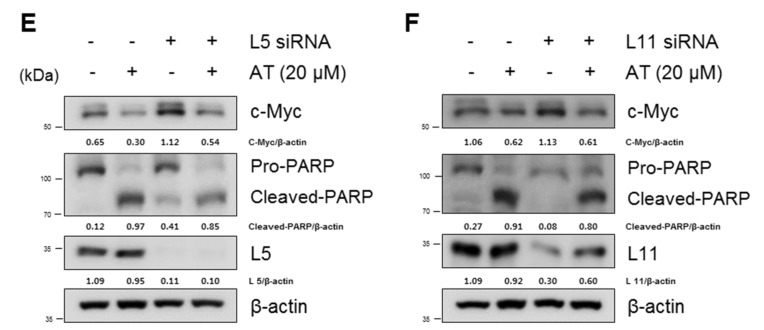
Atorvastatin induced apoptosis via ribosomal protein L5 and L11 in NSCLCs. (**A**) Effect of Atorvastatin on apoptotic bodies in H596, H460, and H1299 cells. The DAPI staining was used to detect apoptotic bodies in H596, H460, and H1299 cells treated with Atorvastatin (10 μM). Arrows indicate apoptotic bodies. Bar scale = 20 μm, DAPI-blue. (**B**) Effect of Atorvastatin on sub G1 population in H1299 cells. H1299 cells were treated with Atorvastatin (0, 5, 10, and 20 μM) for 48 h and stained with propidium iodide (PI) after fixation. Stained cells were analyzed using a FACS Vantage flow cytometry system. (**C**) Effect of Atorvastatin on PARP in H596, H460, and H1299 cells. The cells were exposed to Atorvastatin (10 μM) for 48 h and subjected to Western blotting with antibody of PARP. (**D**) Effect of Atorvastatin on PARP, caspase 3, p53 and β-actin in H596, H460, and H1299 cells. The cells were exposed to Atorvastatin (10 μM) for 48 h and subjected to Western blotting with antibodies of PARP, caspase 3, p53 and β-actin. (**E**,**F**) Effect of Atorvastatin on c-Myc, PARP, L5, L11, and β-actin in H1299 cells transfected with or without L5 siRNA or L11 siRNA plasmid. The cells were exposed to Atorvastatin (20 μM) for 48 h and subjected to Western blotting with antibodies of c-Myc, PARP, L5, L11, and β-actin.

Fluorescein labelled DAPI (blue) staining was used to detect apoptotic bodies. Atorvastatin increased the number of apoptotic bodies in H596, H460, and H1299 cells (Figure 2A). Atorvastatin increased Sub-G1 population as shown in Figure 2B. Consistently, Western blotting was carried out in H596, H460, and H1299 cells. Herein Atorvastatin at 10 μM induced the cleavages of PARP in H596, H460, and H1299 cells (Figure 2C). Also, Atorvastatin (10 and 20 μM) induced cleavages of PARP and caspase3 in H596, H460, and H1299 cells. Of note, p53 phosphorylation was accentuated in in H596 and H460 cells, but not in p53 null type H1299 cells. (Figure 2D). From above data, AT was most sensitive to H1299 cells rather than H596 and H460 cells. Thus, mechanism study was conducted mainly in H1299 cells. Of note, Atorvastatin inhibited the expression of c-Myc and induced ribosomal protein L5 and L11, but depletion of L5 reduced PARP cleavages induced by Atorvastatin than L11, implying the essential property of L5 in Atorvastatin induced apoptosis (Figure 2E,F).

### 2.3. Atorvastatin Induced Autophagy in H596, H460, and H1299 Cells

**Figure 3:**
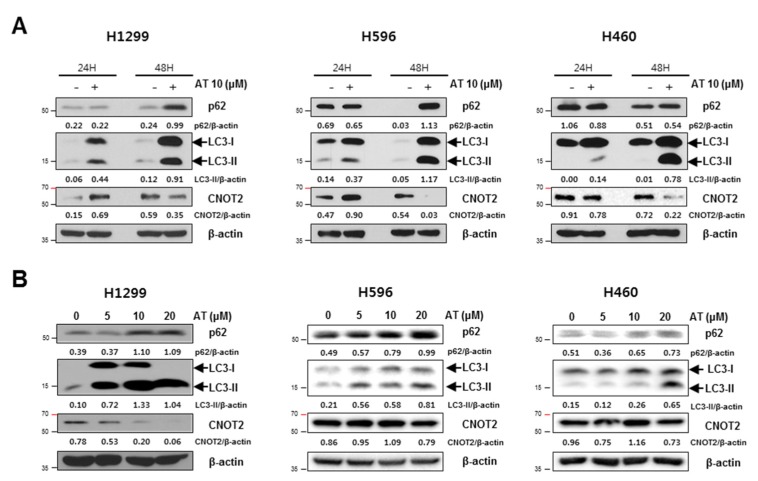

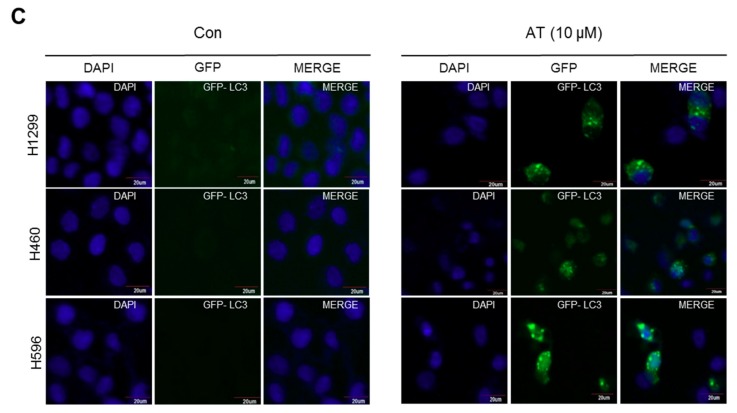
Atorvastatin induces autophagy in H596, H460, and H1299 cells. (**A**) Effect of Atorvastatin on p62/SQSTM 1, LC3I/II, and CNOT2 for 24 h or 48 h in a time course. Western blotting was performed with antibodies of p62/SQSTM 1, LC3I/II, and CNOT2 in Atorvastatin (10 μΜ) treated H596, H460, and H1299 cells. (**B**) Effect of Atorvastatin on p62/SQSTM 1, LC3I/II and CNOT2 in Atorvastatin treated H596, H460, and H1299 cells in a concentration dependent fashion. Three NSCLCs were treated with various concentrations of Atorvastatin (0, 5, 10, and 20 μM) and were subjected to Western blotting with antibodies of p62/SQSTM 1, LC3I/II and CNOT2 in H596, H460, and H1299 cells. (**C**) Effect of Atorvastatin on GFP-LC3 puncta in H1299 cells by Immunofluorescence assay. H596, H460, and H1299 cells were transfected with GFP-fused LC3 plasmid and also exposed to Atorvastatin (10 μM) for 48 h. Then the distribution of GFP-LC3II expression was visualized by confocal microscopy. Bar scale = 20 μm, DAPI-blue, GFP-LC3-Green.

Atorvastatin increased the expression of p62/SQSTM1 and conversion of LC3 I to LC3 II in a time and concentration dependent manner in three NSCLCs. Interestingly, Atorvastatin (10 μM) treatment attenuated the expression of CNOT2 in a time and concentration dependent manner for 48 h in three NSCLCs (Figure 3A,B). Immunofluorescence revealed that GFP-LC3 green fluorescent puncta were observed in cytoplasm of three NSCLCs as an autophagy marker (Figure 3C).

### 2.4. Late Stage Autophagy Inhibitor CQ, but not 3-MA, Enhanced Cytotoxicity and Decreased p62 and Activated LC3II in Atorvastatin Treated H1299 Cells

**Figure 4:**
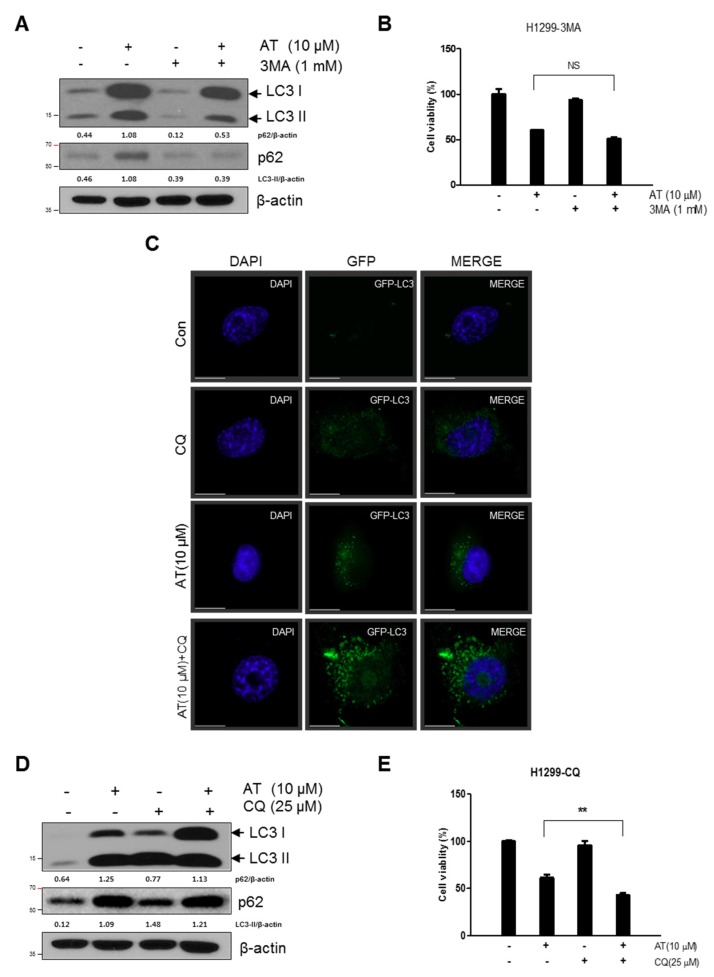
Effect of 3-MA or CQ on Atorvastatin induced-autophagy in H1299 cells. (**A**) Effect of 3-MA on the expression of p62 and LC3II in Atorvastatin treated H1299 cells. (**B**) Effect of 3-MA on cytotoxicity of Atorvastatin treated H1299 cells. (**C**) Effect of the autolysosome inhibitor CQ on GFP- LC3II puncta in Atorvastatin treated H1299 cells. After transient transfection with the GFP-fused LC3 plasmid, H1299 cells were treated with Atorvastatin for 48 h and were incubated with 25 μM CQ for 12 h. The distribution of GFP-LC3II expression was visualized by confocal microscopy. Bar scale = 15 μM, GFP-LC3-Green. (**D**) Effect of CQ on the expression of LC3II and p62 in Atorvastatin treated H1299 cells. Western blotting was performed to determine the expression of LC3II and p62 in H1299 cells treated with Atorvastatin for 48 h. (**E**) Effect of CQ on the cytotoxicity of Atorvastatin treated H1299 cells. H1299 cells were treated with Atorvastatin (10 μM) for 48 h. Cell viability was determined by MTT assay. ** *p* < 0.01 vs. untreated control.

To assess whether LC3 lipidation induced by Atorvastatin treated H1299 cells is due to increased formation of autophagosome or decreased degradation, we measured autophagic flux with CQ which is an inhibitor of acidification that blocks the fusion of lysosome. 3-MA blocked overexpression of p62 and accumulation of LC3II in AT treated H1299 cells (Figure 4A) but, did not affect cytotoxicity (Figure 4B). After transfection of GFP-fused LC3II plasmid, immunofluorescence assay showed that punctuate GFP-LC3II were more accumulated by combination treatment of Atorvastatin and CQ compared to Atorvastatin or CQ alone. (Figure 4C) Consistently, Western blotting showed that combination treatment of Atorvastatin and CQ increased conversion of LC3I into LC3II and p62 expression level (Figure 4D) and cytotoxicity with significance (Figure 4E).

### 2.5. Atorvastatin Induced Impaired Autophagy in NSCLCs

**Figure 5:**
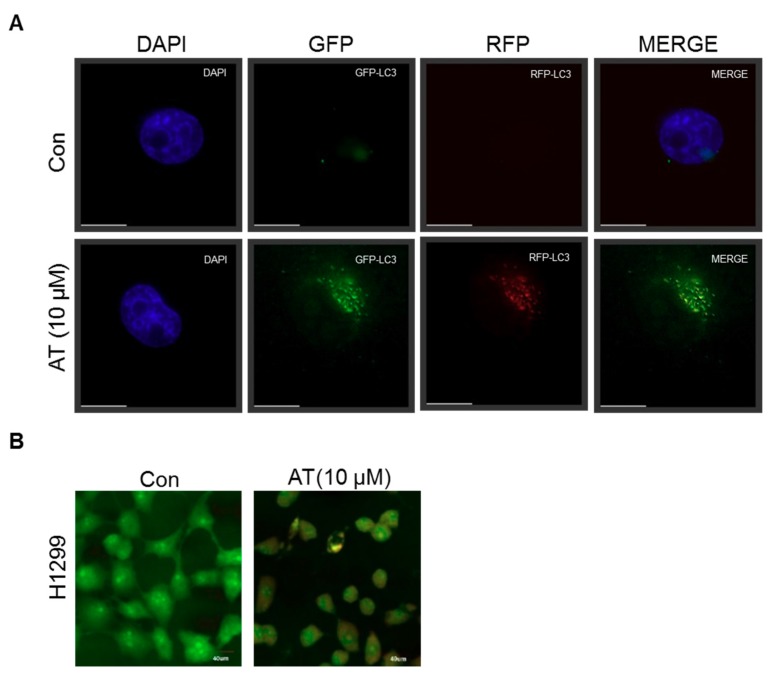
Atorvastatin induces impaired autophagy in H596, H460, and H1299 cells. (**A**) Effect of Atorvastatin on impaired autophagic flux in H1299 cells. H1299 cells were transfected with mRFP-GFP-LC3 constructs, exposed to Atorvastatin (10 μM) for 48 h, and observed by confocal microscope. Bar scale = 15 μm. (**B**) Effect of Atorvastatin on acidic autophagic vacuoles in Atorvastatin treated H1299 cells. H1299 cells were treated with Atorvastatin for 48 h and were stained with acridine orange (AO). Bar scale = 40 μm.

Furthermore, to check whether or not Atorvastatin induces complete autophagy by generating fusion of lysosome and autophagosome, autophagy flux was evaluated in Atorvastatin treated H1299 cells transfected with RFP-GFP-LC3B construct. Here yellow images were shown in Atorvastatin treated H1299 cells transfected with RFP-GFP-LC3B construct (Figure 5A). Likewise, to confirm whether or not Atorvastatin induces late stage autophagy, H1299 cells were stained with acridine orange (AO), which is used for staining acidic vacuoles, including lysosomes, endosomes, and autolysosomes 48 h after Atorvastatin treatment. Similarly, yellowish puncta of GFP-LC3II were observed in H1299 cells, not orange color (Figure 5B) by acridine orange staining and also weak red color was colocalized with the acetotrophic Lysotracker red DND-99 dye in Atorvastatin treated H1299 cells.

### 2.6. Pivotal Role of CNOT2 in Atorvastatin Induced Apoptotic and Apoptotic and Autophagic Cell Death in H1299 Cells

**Figure 6:**
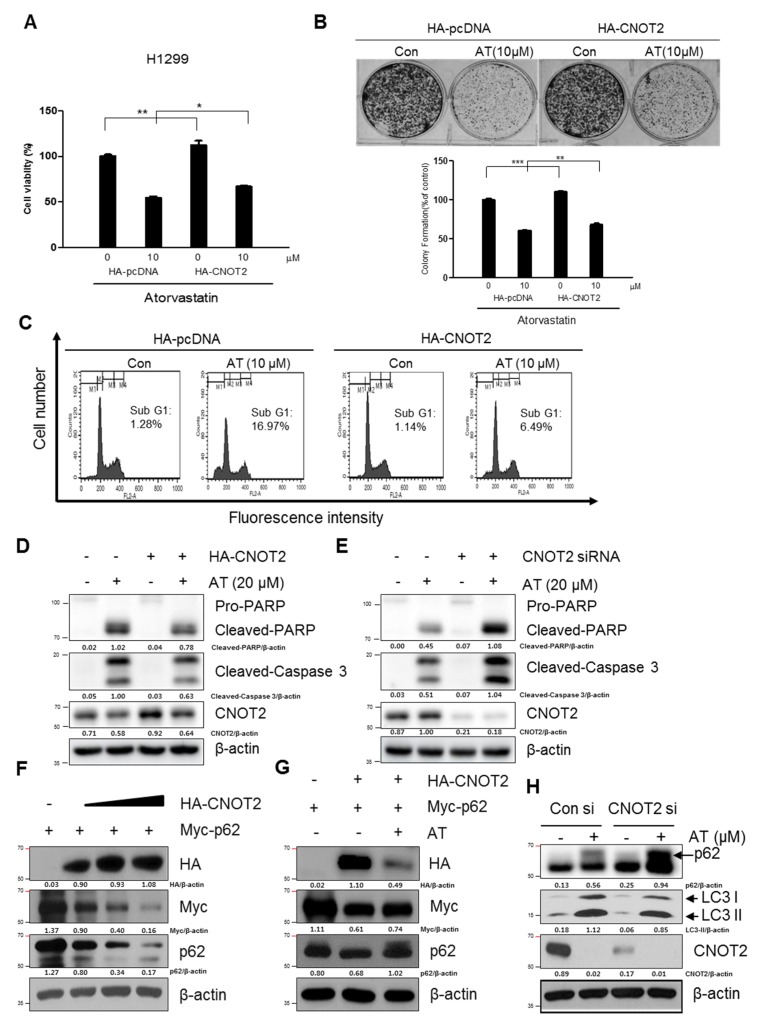
Critical role of CNOT2 in Atorvastatin induced apoptotic and autophagic cell death in H1299 cells. (**A**) Effect of CNOT2 overexpression on cytotoxicity in Atorvastatin treated H1299 cells transfected with HA-CNOT2 plasmid. (**B**) Effect of CNOT2 overexpression on anti-proliferative effect in Atorvastatin (10 μM) treated H1299 cells after transfection with HA-CNOT2 plasmid. (**C**) Effect of CNOT2 overexpression on Sub-G1 population in Atorvastatin (10 μM) treated H1299 cells for 48 h after transfection with HA-CNOT2 plasmid. * *p* < 0.05. ** *p* < 0.01 vs. untreated control. (**D**) Effect of CNOT2 overexpression on cleavages of PARP and caspase3 in Atorvastatin (20 μM) treated H1299 cells transfected with HA-CNOT2 plasmid. (**E**) Effect of CNOT2 depletion on cleavages of PARP and caspase3 in Atorvastatin (20 μM) treated H1299 cells by siRNA transfected with control or CNOT2 siRNAs. (**F**) Effect of CNOT2 overexpression on p62 expression in H1299 cells transfected with HA-CNOT2 plasmid. Western blotting was conducted with antibodies of Myc-p62, p62 and HA-CNOT2 in H1299 cells with or without treatment of Atorvastatin (10 μM). (**G**) Effect of CNOT2 overexpression on p62 accumulation by Atorvastatin in H1299 cells transfected with HA-CNOT2 plasmid. (**H**) Effect of CNOT2 depletion by siRNA transfection on LC3II conversion in H1299 cells transfected with control or CNOT2 siRNA plasmid.

Next, we investigated whether CNOT2 is involved in Atorvastatin induced-apoptosis and autophagy in H1299 cells transfected with HA-CNOT2 plasmid. Overexpression of CNOT2 reduced cytotoxicity and anti-proliferative effects of Atorvastatin (10 μM) treatment for 48 h in H1299 cells (Figure 6A,B). Likewise, CNOT2 overexpression reversed Sub-G1 accumulation and cleavages of PARP and caspase3 in Atorvastatin (20 μM) treated H1299 cells (Figure 6C,D). In contrast, CNOT2 depletion enhanced cleavages of PARP and caspase3 in Atorvastatin (20 μM) treatment for 48 h in H1299 cells (Figure 6E). CNOT2, one of subunits of CCR4-NOT complex, plays a critical role in transcriptional regulation and modulates mRNA degradation [20]. To explore the underlying functional protein-protein interactions between CNOT2 and p62/SQSTM1, the effect of CNOT2 using HA-CNOT2 plasmid was evaluated on p62/SQSTM1 in H1299 cells. Here, CNOT2 overexpression decreased the expression level of p62/SQSTM1 activated by Atorvastatin in H1299 cells (Figure 6F,G). Consistently, CNOT2 depletion enhanced LC3II accumulation induced by Atorvastatin in H1299 cells by using siRNA transfection method (Figure 6H).

## 3. Discussion

In the current study, the underlying antitumor mechanisms of a hypolipidemic agent Atorvastatin as a repurposing drug were examined in H460, H596, H1299, and A549 cells. Here Atorvastatin significantly increased cytotoxicity in H1299, A549, H596, and H460 cells compared with A549 cells. To confirm whether its cytotoxicity is due to apoptotic cell death, DAPI staining and cell cycle analysis were performed in H460, H596, and H1299 cells. Current study showed that Atorvastatin increased sub G1 population and the number of apoptotic bodies as one of apoptotic features, implying the cytotoxicity was mediated by apoptotic cell death.

It is well documented that apoptosis is induced mainly via caspase dependent pathway including mitochondrial and cell death dependent pathways [21]. Also, p53, a tumor suppressor [22,23,24], mediates caspase dependent apoptosis in several cancers [25,26]. Herein Atorvastatin cleaved PARP and caspase 3 in H1299, H596, and H460 cells, but induced p53 phosphorylation only in p53 wild type A549 and H460 cells, but not in p53 null type H1299 cells, indicating caspase dependent apoptosis by Atorvastatin. Considering the endogenous features of p53 null and EGFR^low^ H1299 cells and p53 wild and EGFR^high^ A549 cells [27], the important roles of p53 and EGFR can be supposed based on the results that H1299 cells are more susceptible to Atrovastatin compared with A549 cells. Nonetheless, further study is required to elucidate the different effect of Atrovastatin on p53/MDM2/EGFR and epithelial–mesenchymal transition (EMT) pathway in H1299 cells and A549 cells in vitro and in vivo in the near future.

Previous evidences demonstrate that c-Myc and its regulated ribosomal biogenesis [28] by ribosomal RNA synthesis and processing are critically involved in cancer progression [29,30] and ribosomal protein L5 (RPL5), RPL11, and RPL23 regulates apoptosis in response to ribosomal stress [31,32,33,34]. Herein, it is noteworthy that Atorvastatin inhibited the expression of c-Myc and induced ribosomal protein L5 and L11, but depletion of ribosomal protein L5 reduced PARP cleavages induced by Atorvastatin rather than L11 in H1299 cells, implying important role of L5 in Atorvastatin induced apoptosis.

Autophagy is a physiological dynamic process consisting of three sequential step such as formation of autophagosomes, the fusion of autophagosomes with autolysosomes and lysosomal degradation [35,36]. There are accumulating evidences that incomplete autophagy induction is closely associated with cell death in several cancers [37,38]. Herein, Atorvastatin showed autophagic features by increased expression of the conversion of LC3B-I to LC3B-II, accumulation of p62/SQSTM1, and increased green fluorescent LC3II puncta in NSCLCs, demonstrating autophagic cell death potential of Atorvastatin, since overexpression of p62 is shown in incomplete autophagy, not leading to lysosome fusion and acidification [39].

Accumulating evidences reveal that autophagosome is visualized as yellow or orange puncta (RFP-GFP-LC3 II) in merged images, whereas red puncta (mRFP-LC3II) represents autophagolysosome, since acidification reduces green fluorescence [40]. Herein autophagy flux assay using a tandem fluorescent-tagged LC3 reporter plasmid (mRFP-GFP-LC3) [8,40] and acridine orange-staining [7] showed that Atorvastatin increased the number of yellow-colored LC3 puncta through merging of GFP and mRFP-LC3, not red color, implying incomplete autophagy of Atorvastatin. Consistently, late stage autophagy inhibitor chloroquine (CQ) increased cytotoxicity in Atorvastatin treated H1299 cells compared to early stage autophagy inhibitor 3-MA, showing increased autophagosomes rather than fusion with autophagolysosomes for autophagy associated cell death.

CNOT2, a subunit of CCR4-NOT complex, is known to be involved in proliferation, angiogenesis, apoptosis, metastasis and autophagy in several type of cells [14,16,41,42]. Notably, we found that overexpression of CNOT2 reduced the ability of Atorvastatin to increase cytotoxicity, sub G1 population, cleavages of PARP and caspase 3, LC3II conversion and p62 accumulation in H1299 cells transfected with HA-CNOT2 plasmid. In contrast, CNOT2 depletion enhanced cleavages of PARP and caspase 3, LC3 conversion and p62/SQSTM1 accumulation in H1299 cells, indicating an important role of CNOT2 in Atorvastatin induced apoptosis and autophagic cell death in H1299 cells.

## 4. Materials and Methods

### 4.1. Chemical and Reagents

Atorvastatin calcium salt trihydrate (AT, PZ0001), Chloroquine (CQ, 50-63-5), 3-Methyladenine (3-MA, M9281) were purchased from Sigma Chemical Co. (St. Louis, MO, USA). Also, the antibodies for PARP, Cleaved caspase 3, p62, LC3II, CNOT2, p53, L5, L11, c-Myc (Cell signaling, Beverly, MA, USA), Myc, HA (Santacruz biotechnology, Dellas, TX, USA) and β-actin (Sigma Aldrich Co., St. Louis, MO, USA) were purchased for Western blot analysis.

### 4.2. Cell Culture

Non-small cell lung cancer (NSCLC) cells (H460, H596, A549, H1299) were obtained from American Type Culture Collection (ATCC, University Boulevard Manassas, VA, USA). The cells were cultured at 37 °C in a humidified atmosphere of 95% air and 5% CO_2_ using RPMI 1640 medium (Invitrogene, Carlsbad, CA, USA) supplemented with 10% fetal bovine serum (FBS) (Welgene, Daegu, Korea), penicillin (50 U/ml), and 50 μg/ml streptomycin (Invitrogen, Carlsbad, CA, USA).

### 4.3. Cytotoxicity

To investigate cytotoxicity of Atorvastatin, NSCLC cells (1 × 10⁴ cells) were grown to 80% confluence in 96 well plates. After treatment with various concentrations of Atorvastatin for 1–2 days, cell viability was assessed by 3-(4, 5-dimethylthiazol-2-yl)-2,5-diphenyltetrazolium bromide (MTT) assay (Sigma-Aldrich, St. Louis, MO, USA) based on manufacturer’s instruction. The cells were incubated with an MTT working solution (5 mg/mL in PBS) at 37 °C for 2 h. The optical density (OD) was then measured at 570 nm using a microplate reader (Sunrise, TECAN, Männedorf, Switzerland). Cell viability was calculated as the percentage of control.

### 4.4. Colony Formation Assay

NSCLC cells (1 × 10⁵cells) were seeded in 6-well plates and incubated for 24 h. Next day the cells were exposed to Atorvastatin (0, 5, 10, and 20 μΜ) for 48 h. For crystal violet staining, plates were washed with PBS. The colonies were fixed with 1% glutaraldehyde in PBS and stained by using 0.05% crystal violet in PBS. After taking pictures, the number of colonies were counted.

### 4.5. Cell Cycle Analysis

H1299 cells were treated with Atorvastatin for 48 h and fixed with 75% ethanol at −20 °C. The fixed cells were washed twice by using PBS, resuspended in PBS containing RNase A (1 mg/mL) and incubated for 1 h at 37 °C. Then the cells were stained with 500 μL of propidium iodide (50 μg/mL) for 30 min at room temperature in the dark. Finally, the DNA contents of the stained cells were analyzed using CellQuest Software with the FACS Calibur flow cytometer (Becton Dickinson, Franklin Lakes, NJ, USA).

### 4.6. Apoptosis Detection by DAPI Staining

H1299 cells were exposed to Atorvastatin (10 μM) for 48 h. The cells were fixed in 4% (*v/v*) methanol-free formaldehyde solution. The slides were mounted, and nuclei were counterstanined with DAPI (10 μg/mL) (Sigma, USA) solution for 25 min at room temperature in the dark. The slides were visualized under a FLUOVIEW FV10i confocal microscopy (Olympus, Tokyo, Japan).

### 4.7. Immunoblot Analysis

Total proteins from Atorvastatin treated cells were extracted using lysis buffer (50 mM Tris–HCl, pH 7.4, 150 mM NaCl, 1% Triton X-100, 0.1% sodium dodecyl sulfate (SDS), 1 mM ethylenediaminetetraacetic acid (EDTA), 1 mM Na_3_VO_4_, 1 mM NaF, protease inhibitor cocktail). The proteins were separated on 10–12.5% SDS-PAGE and transferred to the nitrocellulose membrane, a Hybond ECL transfer Membrane (GE Healthcare Bio-Science, Piscataway, NJ, USA). After blocking with 5% nonfat dry milk, the membrane was incubated with the desired primary antibodies of PARP, Cleaved caspase 3, p62, LC3II, CNOT2, p53, c-Myc, L5 and L11 (Cell signaling, Beverly, MA, USA) and β-actin (Sigma Aldrich Co., St. Louis, MO, USA), then followed by a horseradish peroxidase-labeled anti-rabbit or mouse IgG. The immune-reactive bands were visualized by an enhanced chemiluminescence detection kit (Amersham Pharmacia, Piscataway, NJ, USA).

### 4.8. Detection of Acidic Vesicular Organelles

To check the formation of acidic vesicular organelles (AVOs), acridine orange staining (Sigma Chemical Co, St. Louis, MO, USA) was conducted. H1299 cells were stained with 1 μg/mL acridine orange for 15 min and were observed under FLUOVIEW FV10i confocal microscopy (Olympus, Tokyo, Japan).

### 4.9. Autophagic Flux Assay

H1299 cells were transfected with 1 μg of GFP-fused LC3-II or mRFP-GFP-LC3 construct (gifted by prof Hongbo) using Fugene6 (Applied Biosystems, Foster City, CA, USA) transfection reagent. One day later, the cells were exposed to Atorvastatin (10 μM) for 48 h and the distribution of GFP-LC3 and GFP/RFP-LC3 was visualized by a Delta Vision imaging system (Applied Precision. Issaquah, WA, USA).

### 4.10. Immunofluorescence Assay

H1299 cells were exposed to Atorvastatin (10 μM) for 48 h, plated onto poly-l-lysine coated slide glass and fixed in 4% methanol free formaldehyde solution (pH 7.4) at 4 °C for 25 min. The cells were permeabilized in 0.2% Triton X-100, blocked with 5% bovine serum albumin (BSA), 0.5% Tween-20 in a humidified chamber and incubated with the LC3 antibody (Cell signaling, Danvers, MA, USA.). The slides were mounted with mounting medium with DAPI (Vector Laboratories, Inc., Burlingame, CA, USA) and visualized under an FLUOVIEW FV10i confocal (Olympus, Tokyo, Japan).

### 4.11. Detection of Acidic Vesicular Organelles

To check the formation of acidic vesicular organelles (AVOs), acridine orange staining (Sigma Chemical Co, St. Louis, MO, USA) was conducted. H1299 cells were stained with 1μg/ml acridine orange for 15 min and were observed under FLUOVIEW FV10i confocal microscopy (Olympus, Tokyo, Japan).

### 4.12. RNA Interference

H1299 cells were transfected with siRNA oligoribonucleotides targeted against human CNOT2 or a negative control. The cells were incubated for 48 h with 1 nM of siRNA using INTERFERIN siRNA transfection reagent (Polyplus-transfection Inc., New York, NY, USA) according to the manufacturer’s protocol. The cells were then washed off from the plates and transferred into serum-free medium for experiments

### 4.13. Statistical Analysis

The results were expressed as means ± SD from at least three independent experiments. Student’s *t* test was performed for two group comparisons. In addition, the one-way analysis of variance (ANOVA) followed by a Turkey post hoc test was conducted for multi-group comparison using Graphed Prism software (Version 5.0, La Jolla, CA, USA). Significant difference was considered of the *p* value was less than 0.05.

## 5. Conclusions

Through several experiments to explore the underlying antitumor mechanism of a hypolipidemic agent, Atorvastatin increased cytotoxicity, sub G1 population, the number of apoptotic bodies, cleaved PARP and caspase 3, activated p53 in H1299, H596 and H460 cells and also induced LC3 II conversion, p62 SQSTM1 accumulation with increased number of LC3II punctae in H1299 cells. However, CQ enhanced cytotoxicity in Atorvastatin treated H1299 cells compared to 3-MA with impaired autophagic flux. Notably, overexpression of CNOT2 reversed the ability of Atorvastatin to increase cytotoxicity, sub G1 population, cleavages of PARP and caspase 3, LC3II conversion and p62/SQSTM1 accumulation in H1299 cells. However, CNOT2 depletion potentiated cleavages of PARP and caspase 3, LC3 conversion and p62/SQSTM1 accumulation by Atorvastatin in H1299 cells. Overall, these findings support evidences that CNOT2 signaling is critically involved in Atorvastatin exerted antitumor effect in NSCLCs.
